# C9ORF72: What It Is, What It Does, and Why It Matters

**DOI:** 10.3389/fncel.2021.661447

**Published:** 2021-05-05

**Authors:** Julie Smeyers, Elena-Gaia Banchi, Morwena Latouche

**Affiliations:** ^1^Sorbonne Université, Institut du Cerveau - Paris Brain Institute - ICM, Inserm, CNRS, APHP, Hôpital de la Pitié Salpêtrière, DMU Neuroscience 6, Paris, France; ^2^PSL Research university, EPHE, Neurogenetics team, Paris, France

**Keywords:** *C9ORF72*, FTD/ALS, loss-of-function, autophagy, inflammation

## Abstract

When the non-coding repeat expansion in the *C9ORF72* gene was discovered to be the most frequent cause of frontotemporal dementia (FTD) and amyotrophic lateral sclerosis (ALS) in 2011, this gene and its derived protein, C9ORF72, were completely unknown. The mutation appeared to produce both haploinsufficiency and gain-of-function effects in the form of aggregating expanded RNAs and dipeptide repeat proteins (DPRs). An unprecedented effort was then unleashed to decipher the pathogenic mechanisms and the functions of C9ORF72 in order to design therapies. A decade later, while the toxicity of accumulating gain-of-function products has been established and therapeutic strategies are being developed to target it, the contribution of the loss of function starts to appear more clearly. This article reviews the current knowledge about the C9ORF72 protein, how it is affected by the repeat expansion in models and patients, and what could be the contribution of its haploinsufficiency to the disease in light of the most recent findings. We suggest that these elements should be taken into consideration to refine future therapeutic strategies, compensating for the decrease of C9ORF72 or at least preventing a further reduction.

## Introduction

Frontotemporal dementia (FTD) and amyotrophic lateral sclerosis (ALS) are rare neurodegenerative diseases characterized by behavioral and motor disorders, respectively. FTD results from the degeneration of the frontal and/or temporal cortical lobes, resulting in cognitive and/or language impairment (Gorno-Tempini et al., 2011; Rascovsky et al., 2011). ALS is the most frequent motor neuron disease. It is caused by the degeneration of upper motor neurons and their corticospinal axonal tracts (lateral sclerosis) associated with the loss of lower motor neurons and their axons, which leads to muscle wasting (amyotrophy) and paralysis (Mitsumoto et al., 1998). Despite their different symptomatology, the report of some overlap between these diseases can be traced as far back as 1869 (Charcot, 1869) and 1892 (Marie, 1892) and has progressively been more and more documented over the years (Lomen-Hoerth et al., 2002, 2003; Lillo and Hodges, 2009). It is now recognized that FTD and ALS are part of a clinical, neuropathological, and genetic continuum (Ling et al., 2013; Burrell et al., 2016). This interrelation was particularly strengthened when TDP-43 was discovered in 2006 to be the main ubiquitinated protein composing the major form of neuropathological aggregates in both FTD and ALS (Arai et al., 2006; Neumann et al., 2006). Moreover, 2006 was marked by the identification, on chromosome 9p, of the main locus associated with familial forms of FTD/ALS, the defining term for the cases associating clinical features of both FTD and ALS (Morita et al., 2006; Vance et al., 2006). The quest for the corresponding causal gene and mutations lasted 5 years and ended with the identification of a hexanucleotide (G_4_C_2_)*_n_* repeat expansion (HRE) in the non-coding region of chromosome 9 open reading frame 72, *C9ORF72* (DeJesus-Hernandez et al., 2011; Renton et al., 2011; Gijselinck et al., 2012). Until then, this gene and the protein of the same name had not been studied, but the need to unravel the etiopathogenesis of C9-FTD/ALS has triggered a massive effort in studying the function of the C9ORF72 protein and its relevance in disease. Therefore, we focus this review on what is now known about C9ORF72.

The HRE in *C9ORF72* is the most frequent mutation in familial FTD, ALS, and FTD/ALS (Majounie et al., 2012; van Blitterswijk et al., 2012; Gijselinck et al., 2016). Patients are heterozygous and eventually all carriers develop the disease, but the age at onset is highly variable, from mid-20s to the ninth decade. Therefore, the penetrance is better defined as age-related and goes from 50 at 58 years of age to 99.5% by 83 years of age (Murphy et al., 2017). Age at onset variability is common in repeat expansion disorders and is generally correlated to the size of the expansion, but in the case of C9-FTD/ALS, this is a matter of intense debate as positive (Beck et al., 2013; Hübers et al., 2014; Waite et al., 2014; Fournier et al., 2019) and negative (Gijselinck et al., 2016) correlations as well as absence of a correlation (Dols-Icardo et al., 2014; Suh et al., 2015; Chen et al., 2016; Jackson et al., 2020) were reported. The non-coding G_4_C_2_ expansion has two main consequences: a loss-of-function effect causing C9ORF72 haploinsufficiency and a gain of function associated with the expression of abnormal bidirectionally transcribed RNAs carrying the repeat (DeJesus-Hernandez et al., 2011). These expanded RNAs accumulate in RNA foci or happen to be translated into dipeptide repeat proteins (DPRs) *via* repeat-associated non-ATG (RAN) translation (Ash et al., 2013; Lagier-Tourenne et al., 2013; Mizielinska et al., 2013; Mori et al., 2013). Pathologically, *C9ORF72* expansion carriers present TDP-43 inclusions in neurons and oligodendroglial cells that do not overlap with the neuronal DPRs and RNA foci in neurons and glial cells (Mizielinska et al., 2013; Mackenzie et al., 2014). After a decade of research and the generation of a number of informative model systems, the understanding of the contribution of the different gain-of-function and loss-of-function mechanisms in the pathogenesis is still blurry (Balendra and Isaacs, 2018; Braems et al., 2020). However, recent results strongly suggest that the combination of the loss-of-function effect with some gain-of-function entities is essential for the development of a pathological FTD/ALS phenotype (Shi et al., 2018; Shao et al., 2019; Lutz, 2020; Zhu et al., 2020). Here, we review what is known about the C9ORF72 protein, how it is affected in C9-FTD/ALS, and explore the possible contribution of haploinsufficiency to the disease pathogenesis.

## C9ORF72 Physiological Functions

### Gene Architecture, Transcripts, and Protein Distribution

**FIGURE 1:**
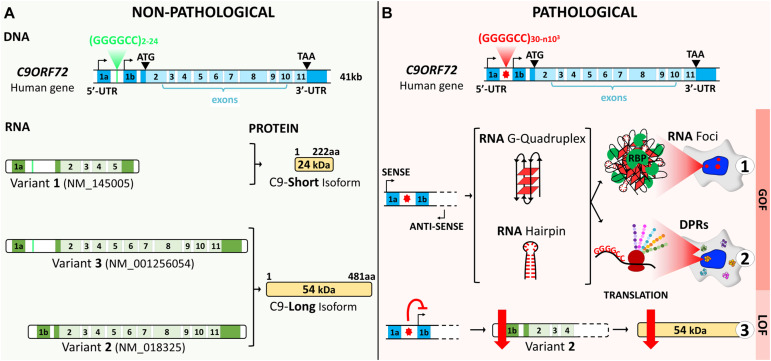
*C9ORF72* gene structure, transcript variants, and protein isoforms under a non-pathological **(A)** and a pathological **(B)** state. **(A)** The *C9ORF72* human locus includes two non-coding exons (1a and 1b) and 10 coding exons (from 2 to 11). It gives rise to three coding variants: variant 1, *V1*, which includes exon 1a and exons 2–5; variant 2, *V2*, which includes exon 1b and exons 2–11; and variant 3, *V3*, which includes exon 1a and exons 2–11. Alternative splicing of these three RNA variants results in the production of two different isoforms: the 222-amino acid (aa) isoform (C9-short of 24 kDa) encoded by V1 and the 481-aa isoform (C9-long of 54 kDa) encoded by V2 and V3. The coding exons are indicated in *light blue* and the non-coding exons in *dark blue*. **(B)** In a pathological state, the G_4_C_2_ repeat localized between the two non-coding exons (1a and 1b) is abnormally expanded and results in three possible pathogenic mechanisms. Bidirectional transcription of the hexanucleotide repeat expansion (HRE) generates G_4_C_2_ sense and G_2_C_4_ antisense expanded RNAs. These HRE transcripts give rise to G-quadruplex and hairpin structures that can form RNA foci and sequester RNA-binding proteins (*RBP*) (*B1*). Expanded RNAs are also translated through repeat-associated non-ATG (*RAN*) translation, resulting in the synthesis of dipeptide protein repeats (*DPRs*) (*B2*). Finally, the presence of the HRE inhibits transcription, leading to a decrease in the C9ORF72 protein (*B3*).

The human chromosome 9 open reading frame 72 (*C9ORF72*) locus is located on the reverse strand of chromosome 9 (41 kb). It includes two non-coding exons (1a and 1b) and 10 coding exons (from 2 to 11) and gives rise to three coding variants (Figure 1A). Variant 1, V1 (NM_145005), is a short transcript including non-coding exon 1a and exons 2–5 as the coding sequence. V2 (NM_018325) and V3 (NM_001256054) differ in their inclusion of the non-coding exon 1b or 1a, respectively, and share exons 2–11 as the coding sequence. Alternative splicing of these three RNA variants results in the production of two different isoforms: the 222-amino acid (aa) isoform (C9-short of 24 kDa) encoded by V1, while the 481-aa isoform (C9-long of 54 kDa) is encoded by V2 and V3 (Figure 1A; DeJesus-Hernandez et al., 2011; Renton et al., 2011; Gijselinck et al., 2012). The *C9ORF72* human gene is highly conserved in primates and across different species commonly used as model systems, suggesting that the protein(s) encoded by *C9ORF72* have fundamental biological functions. The similarity between the sequences of the orthologous genes and the human gene, expressed as a percentage of identity, is very high in chimpanzee (gene LOC465031, 99.58%), rhesus macaque (gene C15H9orf72, 99.58%), mouse (gene *311004O21Rik*, 98.13%), rat (gene RGD1359108, 97.71%), rabbit (gene *C9orf72*, 98.54%), *Xenopus* (gene *C9orf72*, 83.96%), and zebrafish (gene *C13H9orf72*, 75.97%). However, for the nematode, the similarity is very poor (*Caenorhabditis elegans*, gene F18A1.6, 14.71%), and there is no ortholog of *C9orf72* in *Drosophila* (query > *C9ORF72* human gene)^1^.

In humans, *C9ORF72* transcripts are detectable in most tissues, and notably in all brain regions and the spinal cord (DeJesus-Hernandez et al., 2011; Renton et al., 2011; Rizzu et al., 2016). Regarding cell type distribution, *C9ORF72* expression is particularly high in myeloid cells (in particular in CD14^+^ monocytes, eosinophils, and neutrophils) and lower in lymphoid cells and other tissues (Rizzu et al., 2016). Similarly, in mice harboring a LacZ reporter instead of exons 2–6 of the endogenous *C9orf72* gene, the activity of the *C9orf72* promoter is found in several tissues (lung, heart, liver, spleen, and kidney muscle), with the highest levels in the brain and spinal cord (Suzuki et al., 2013; Atanasio et al., 2016; Jiang et al., 2016; Langseth et al., 2017). This large expression profile was confirmed by *in situ* hybridization for the mouse *C9orf72* ortholog (Koppers et al., 2015). LacZ expression in the central nervous system (CNS) initially appeared restricted to neurons (Suzuki et al., 2013), but later reports demonstrated that some expression could be found also in glial cells (Jiang et al., 2016; Langseth et al., 2017). Transcriptomic analyses have further demonstrated that, in mice, as in humans, myeloid cells including the microglia, macrophages, and dendritic cells (CD11b^+^) show a high expression of *C9orf72* compared to other immune cell populations such as T (CD3^+^) and B (CD19^+^) cells (Atanasio et al., 2016; O’Rourke et al., 2016). To understand the regulation of *C9ORF72* expression between CNS tissues and cell types, some authors (Rizzu et al., 2016) surveyed the *C9ORF72* locus using cap analysis of gene expression sequence (CAGEseq). They described several novel transcription start sites (TSS) at the *C9ORF72* human locus, both in the sense and antisense strands. These TSS have distinct modes of expression in myeloid cells, lymphoid cells, and tissues from the CNS (cerebellum, cortex tissues, hippocampus, caudate, and putamen), suggesting they have cell type- and/or tissue-specific functions (Rizzu et al., 2016). Concurrently, at least five long non-coding RNAs (lncRNAs), two sense and three antisense lncRNAs, along the *C9ORF72* gene have been described that arise from the same promoter region as the sense *C9ORF72* coding transcripts (Rizzu et al., 2016). The functions of these *C9ORF72* lncRNAs are yet unknown, but they might participate in the transcriptional regulation of *C9ORF72* (Douglas, 2018).

At the protein level, it is important to highlight that the range and specificity of available antibodies have dramatically evolved, allowing the community to progress from initially confusing results to a much better understanding of the C9ORF72 protein expression profile and subcellular localization (Atkinson et al., 2015; Xiao et al., 2015; Ferguson et al., 2016; Davidson et al., 2018; Frick et al., 2018; Laflamme et al., 2019). Globally, the C9ORF72 protein is expressed mainly in the brain, spinal cord, and the immune system, and at lower levels in other organs (lung, heart, liver, kidney, and skeletal muscle), in agreement with the expression profile of the transcript. The 481-aa C9-long isoform is the most abundant (Frick et al., 2018). In mouse tissues and the human brain, C9ORF72 appears cytoplasmic, with punctate staining in neurites suggestive of synaptic terminals (Atkinson et al., 2015; Ferguson et al., 2016; Frick et al., 2018; Laflamme et al., 2019; Xiao et al., 2015). Isoform-specific antibodies were developed (Xiao et al., 2015) that showed C9-long to be diffuse in the cytoplasm with some speckle-like structures in neurites, as previously reported. Meanwhile, C9-short appeared localized at the nuclear membrane in postmortem human brain tissue. Other studies have reported that the distribution of C9ORF72 is dynamic in the developing and adult mouse brain, switching between predominantly cytoplasmic and a nucleocytoplasmic distribution according to the developmental stage and the examined isoforms (Atkinson et al., 2015; Ferguson et al., 2016). C9ORF72 was detected in synaptosome preparations from mouse brains and co-localized with synaptic markers in mouse brain tissues and human induced pluripotent stem cell (iPSC)-derived motor neurons (Atkinson et al., 2015; Ferguson et al., 2016; Frick et al., 2018), supporting a role for C9ORF72 at the synapses. Further studies using tagged C9ORF72, transfected or CRISPR modified, or immunocytochemical detection of endogenous C9ORF72 in cell lines or human iPSC-derived neurons have revealed the co-localization of C9ORF72 with various organelles: the Golgi apparatus (Aoki et al., 2017), stress granules (Maharjan et al., 2017; Chitiprolu et al., 2018), mitochondria (Wang et al., 2021), and, most of all, compartments of the endolysosomal pathway (Farg et al., 2014; Sellier et al., 2016; Amick et al., 2016; Frick et al., 2018; Shi et al., 2018; Wang et al., 2020). Recently, an extensive study comparing the 14 commercially available antibodies revealed that many of these antibodies that were used in previous studies fail to properly detect C9ORF72 (Laflamme et al., 2019). Using antibodies with verified selectivity, the authors demonstrated that C9ORF72 was localized in the lysosome, confirming their immunocytochemical results with lysosomal immunoprecipitation. Interestingly, the authors also found that, in the cell lines they used, only 15% of the C9ORF72 protein pool was associated with lysosomes, 25% in total being membrane associated and 75% remaining in the soluble/cytosolic fraction. Moreover, in human monocyte-derived macrophages, where C9ORF72 is strongly expressed, it was localized to late phagosomes and phagolysosomes. Although some constants start to appear, the factors driving the subcellular localization of C9ORF72 in the different cell types and/or the cell states remain to be elucidated.

### C9ORF72 Protein Partners and Interactors

When the mutation of *C9ORF72* was discovered in FTD and ALS cases, nothing was known about the role of the C9ORF72 protein. The accumulating evidence that haploinsufficiency was one of the consequences of the HRE (DeJesus-Hernandez et al., 2011; Gijselinck et al., 2012; Waite et al., 2014; Xiao et al., 2015, 2016; Saberi et al., 2018; Viodé et al., 2018) has triggered an effort to decipher the functions of C9ORF72. Through the characterization of C9ORF72 subcellular distribution, bioinformatics studies, and the identification of protein interactors, some functions of C9ORF72 began to be unraveled.

#### Interactions With the Nuclear Membrane, Cytoskeleton, Mitochondria, and Membrane-Less Processes

Following the discovery of the localization of C9ORF72 at the nuclear membrane, co-immunoprecipitation and immunofluorescence experiments suggested that C9ORF72 interacted with Importin b1 and Ran-GTPase, major proteins involved in nucleocytoplasmic import (Xiao et al., 2015). C9ORF72 also appears to regulate stress granule (SG) formation and degradation (Maharjan et al., 2017; Chitiprolu et al., 2018). C9-long is recruited to SG upon stress-related stimuli. Without C9ORF72, these granules cannot form and other SG-associated proteins like TIA-1, G3BP1, and HuR are downregulated (Maharjan et al., 2017). C9ORF72 is also necessary for cellular recovery after the removal of stress as it associates with p62 to target SG for the degradation by autophagy (Chitiprolu et al., 2018). Through interaction with the cytosolic chaperone Hsc70 (heat shock cognate protein 70), C9ORF72 may also be involved in chaperone-mediated autophagy, or aggrephagy, a mechanism that permits the clearance of aggregating proteins without requiring the presence of autophagic vesicles (Sellier et al., 2016).

**FIGURE 2:**
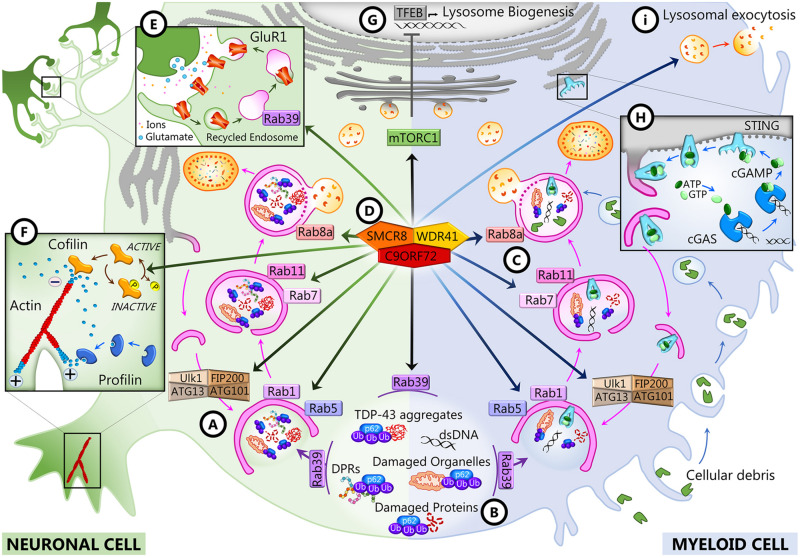
C9ORF72 functions in neurons and myeloid cells. In both cell types, C9ORF72 regulates autophagy and vesicular trafficking at different levels: the initiation of autophagy **(A)**, the recruitment of substrates for autophagy degradation **(B)**, and autophagosome maturation and closure **(C)**. These and other functions depend on the interaction of C9ORF72 with its partners SMCR8 and WDR1 inside a GTPase-interacting complex **(D)**, which is also responsible for interfering with mTORC1 signaling **(G)**. In neurons, C9ORF72 regulates actin dynamics **(F)** and endosomal recycling of GluR1 at the synapse **(E)**. In myeloid cells, C9ORF72 modulates the STING pathway **(H)** and lysosomal exocytosis **(I)**.

A role for C9ORF72 in axon growth was identified in primary mouse embryonic motor neurons, where C9ORF72 overexpression led to longer axons and increased growth cone size, whereas its knockdown resulted in reduced axonal length and smaller growth cones (Sivadasan et al., 2016). The study of C9ORF72 interactome by mass spectrometry-based proteomics identified cofilin and other actin binding proteins such as Arp2, Arp3, and coronin as partners of C9ORF72. Cofilin is an actin binding protein that regulates actin dynamics, depolymerizing the tail of the actin filaments to supply actin monomers ready for polymerization at the growing end of the filament (Lappalainen and Drubin, 1997). It is inactivated by phosphorylation by LIM kinases (Lawler, 1999). Remarkably, cofilin phosphorylation was increased in the absence of C9ORF72 in mouse motor neurons and in brains of C9-FTD/ALS patients compared to controls (Figure 2F; Sivadasan et al., 2016). Actin filament assembly and disassembly are necessary for axonal maintenance and synaptic strength (Flynn et al., 2012; Huang et al., 2013); thus, the alteration of this dynamic may account for the axonal phenotype observed in C9ORF72-depleted mouse motor neurons and iPSC-derived motor neurons from C9-ALS patients (Sivadasan et al., 2016). Neuronal activity and synaptic function also depend on the energy supply provided by mitochondrial oxidative phosphorylation (OXPHOS) complexes (Rangaraju et al., 2019), and C9ORF72 was very recently shown to regulate the activity of these complexes (Wang et al., 2021). By interacting with AIFM1, C9ORF72 is imported to the mitochondrial intermembrane space, where it interacts with TIMMDC1 and the prohibitin (PHB) complex to stabilize OXPHOS complex I (CI) subunits to allow their assembly. In the absence of C9ORF72 as well as in iPSC-derived motor neurons from C9-ALS patients, the level of mature CI and CI activity were reduced (Wang et al., 2021).

#### C9ORF72 Is a DENN Protein Regulating Rab-GTPases

Bioinformatics studies revealed that C9ORF72 shows homology to the DENN (differentially expressed in normal and neoplasia) family of proteins (Yoshimura et al., 2010; Marat et al., 2011; Levine et al., 2013). DENN proteins comprised an N-terminal longin domain, followed by DENN and C-terminal alpha domains (Zhang et al., 2012). These three domains encompass aa 23–150, 212–312, and 313–481 of the human C9-long isoform (Xiao et al., 2016). The C9-short isoform contains only the longin domain, which is associated with the regulation of endomembrane trafficking and contains a region for binding small GTPases (De Franceschi et al., 2014). DENN domain proteins tend to be guanosine diphosphate (GTP)–guanosine diphosphate (GDP) exchange factors (GEFs) for Rab GTPases, which are master regulators of membrane trafficking. Rab proteins are small GTPases that are activated when GTP-bound and inactive when GDP-bound. They interact with GEFs that trigger the binding of GTP, which switches Rab GTPases from the inactive to the active state and allows them to be recruited to membranes (Guadagno and Progida, 2019; Yoshimura et al., 2010). C9ORF72 has been reported to interact with several Rabs to regulate endosomal trafficking, lysosomal biogenesis, and autophagy in cell culture models (Farg et al., 2014; Sellier et al., 2016; Tang, 2016; Webster et al., 2016; Shi et al., 2018). In neuronal cell lines and primary neuronal cultures, C9ORF72 co-localizes and co-immunoprecipitates with some Rabs implicated in autophagy and endosomal transport, including Rab1, Rab5, Rab7, and Rab11, and co-localizes with Rab7 and Rab11 in human motor neurons from the spinal cord (Farg et al., 2014). Depletion of C9ORF72 using siRNA inhibited endocytosis and caused the accumulation of autophagosomes (Farg et al., 2014). In induced motor neurons (iMNs) derived from the iPSCs of C9-FTD/ALS patients, C9ORF72 localizes to Rab5-positive early endosomes, and neuronal death observed in patients’ iMNs was rescued when Rab5 activity was increased (Shi et al., 2018). All these Rabs are at the intersection of endocytosis and macroautophagy (Nassif et al., 2017; Shi et al., 2017; Guadagno and Progida, 2019). Rab1 is involved in endoplasmic reticulum (ER)–Golgi transport, and Rab5 is necessary for endocytosis and is present at early endosomes, but these two Rabs also regulate the initiation of autophagy (Figure 2A). Rab11 associates with recycling endosomes and is involved in autophagosome elongation, while Rab7 associates with late endosomes, is involved in autophagosome maturation and closure, and drives the fusion of late endosomes and autophagosomes to lysosomes (Figure 2C). The interaction of C9ORF72 and Rab7L1 was also shown to regulate extracellular vesicle secretion (Aoki et al., 2017). It was further shown that C9ORF72 interacts with the Unc-51-like kinase 1 (ULK1) autophagy initiation complex and Rab1a and controls the initiation of autophagy by acting as a Rab1a effector (Webster et al., 2016). C9ORF72 binds to the activated ULK1 and recruits active Rab1a to the ULK1 complex to promote translocation of the ULK1 complex to the phagophore during autophagy initiation (Figure 2A). As a consequence, the translocation of ULK1 is impaired when C9ORF72 is knocked down in cell lines and primary neurons and autophagy is markedly reduced in neurons induced from C9-FTD/ALS patients’ iPSCs (Webster et al., 2016).

#### C9ORF72 Forms a Tripartite Complex With SMCR8 and WDR41

The interaction of C9ORF72 with the autophagy initiation complex and with a number of Rabs may be dependent on other C9ORF72 interacting partners, as there is compelling evidence that C9ORF72 forms a tripartite trimer with other DENN domain-containing proteins, SMCR8 (Smith–Magenis chromosome region 8) and WDR41 (WD40-repeat containing protein 41) (Figure 2D; Amick et al., 2016; Blokhuis et al., 2016; Sellier et al., 2016; Sullivan et al., 2016; Ugolino et al., 2016; Yang et al., 2016). This complex was reported to act as GEF for Rab8a (Sellier et al., 2016) and Rab39b (Sellier et al., 2016; Yang et al., 2016) and to interact with the proteins of the autophagy initiation complex FIP200/Ulk1/ATG13/ATG101 to control autophagic flux (Figure 2A; Sellier et al., 2016; Sullivan et al., 2016; Yang et al., 2016). The interaction of the complex with Rab8a and Rab39b also mediates the recruitment of p62 (Sellier et al., 2016), the autophagy receptor that targets poly-ubiquitinated proteins for autophagy degradation (Figure 2B). Interestingly, there is a significant decrease of expression of postsynaptic Rab39b concomitant with the upregulation of GluR1 in *C9orf72* knockout mice forebrains (Xiao et al., 2019), which may be linked with the role of Rab39b in coordinating AMPA receptor subunit (GluR1–4) composition and trafficking to the postsynaptic membrane (Figure 2E; Giannandrea et al., 2010; Mignogna et al., 2015). Increased glutamate receptor expression has also been observed in postmortem cortex and spinal cord of C9-FTD/ALS patients and in iMNs derived from iPSCs from C9 patients, possibly leading to glutamate excitotoxicity (Selvaraj et al., 2018; Shi et al., 2018).

The structure of the C9ORF72–SMCR8–WDR41 (CSW) complex was recently described by Cryo-EM analysis (Su et al., 2020; Tang et al., 2020a, b). The complex is a homodimer of the CSW trimer, and its structure suggested that it can act as a GTPase-activating protein (GAP) rather than a GEF. Indeed, the CSW complex, but not C9ORF72 or SMCR8 alone, was shown to be a GAP for Rab8a and Rab11a (Tang et al., 2020a, b). Similarly, the CSW complex appears to be a GAP for ARF1 and ARF6, previously identified as interactors of C9ORF72 (Sivadasan et al., 2016), and other members of the ADP-ribosylation factor (ARF) family of small GTPases (Su et al., 2020). The ARF small GTPases regulate vesicular traffic and organelle structures (D’Souza-Schorey and Chavrier, 2006; Sztul et al., 2019). Notably, ARF6 regulates actin dynamics (D’Souza-Schorey and Chavrier, 2006; Sivadasan et al., 2016; Sztul et al., 2019), and ARF1 has been reported to promote mTORC1 activation (Jewell et al., 2015). Both processes are altered when C9ORF72 or SMCR8 is depleted (Figures 2F,G; Amick et al., 2016; Sivadasan et al., 2016; Ugolino et al., 2016; Shao et al., 2020; Wang et al., 2020).

Altogether, the analysis of C9ORF72 functions and protein partners indicates that C9ORF72 is a modulator of the activity of small GTPases that regulate membrane trafficking, autophagy, and axonal and synaptic maintenance. Investigating whether SMCR8 and WDR41 and the GAP or GEF activities of the CSW trimer are involved in the other functions of C9ORF72, like the regulation of mitochondrial OXPHOS complexes, will be necessary to complete our understanding of C9ORF72 cellular functions.

### C9ORF72 Regulation of Inflammation

Since 2016, numerous reports have established that C9ORF72 plays an important role in immune regulation. Transcriptomic studies first showed that *C9ORF72* transcripts are mostly expressed in myeloid cells, particularly in CD14^+^ monocytes in human and mice (Rizzu et al., 2016). In particular, C9ORF72 expression is elevated in dendritic and microglial cells (Su et al., 2014; O’Rourke et al., 2016). A genome-wide synthetic lethal screen was therefore conducted in human myeloid cells lacking C9ORF72 to identify its genetic interactors (Chai et al., 2020). That screen identified FIS1, a mitochondrial membrane protein involved in mitochondrial fission and mitophagy, as a strong genetic interactor of C9ORF72. Interestingly, this interaction could be validated also for SMCR8 and WDR41 and was shown to be independent of FIS1 mitochondrial localization and role in mitochondrial fission and mitophagy. In fact, C9ORF72 and FIS1 were both shown to interact with ligands of the receptor for advanced glycation end (RAGE), which is involved in modulating inflammation and chemotaxis. C9ORF72 and FIS1 could work in parallel pathways to repress the secretion of inflammatory cytokines by preventing the binding of these ligands to RAGE (Chai et al., 2020). Interestingly, all *C9orf72* knockout mouse models strikingly develop immune abnormalities and autoimmune-like diseases (Koppers et al., 2015; Atanasio et al., 2016; Burberry et al., 2016; Jiang et al., 2016; O’Rourke et al., 2016; Sudria-Lopez et al., 2016; Sullivan et al., 2016; Ugolino et al., 2016). Indeed, mutant mice present massive macrophages and lymphocyte tissue engorgement, causing splenomegaly, lymphadenopathy, and glomerulonephropathy, accompanied by excessive secretion of inflammatory cytokines and autoantibodies (Atanasio et al., 2016; Burberry et al., 2016; O’Rourke et al., 2016; Sudria-Lopez et al., 2016). Depending on the research facilities where the knockout mice were studied, they presented various degrees of mortality, and some of them died prematurely (Atanasio et al., 2016; Burberry et al., 2016; Sudria-Lopez et al., 2016) and presented an associated motor phenotype (Atanasio et al., 2016). A comprehensive analysis of the environmental differences that could elicit such differences was undertaken by Eggan and colleagues (Burberry et al., 2020). It revealed that C9ORF72 was necessary to maintain immune cell homeostasis in response to immune-stimulating gut microbiota. All phenotypic variations between mice could be abolished using fecal material transplantation, and lifelong suppression of gut microflora with antibiotics could considerably alleviate inflammatory and autoimmune phenotypes in *C9orf72* knockout (KO) mice. The same immune defects, including immune organ hypertrophy, were observed in two mouse lines with a C9ORF72 depletion specifically in myeloid cells (McCauley et al., 2020), confirming that these are the main cells involved in C9ORF72 immune functions. In *C9orf72*fl/fl;*Cx3cr1*Cre or *C9orf72*fl/fl;*LyzM*Cre mice, dendritic cells, in particular among myeloid cells, showed the upregulation of type I interferons through the STING (stimulator of interferon genes) pathway in response to cytosolic DNA (McCauley et al., 2020). STING signaling is regulated by the autophagolysosomal pathway, and blocking its degradation leads to sustained IFN-I production (Konno et al., 2013; Gonugunta et al., 2017; Prabakaran et al., 2018). Due to the role of C9ORF72 in autophagy, it is possible that C9ORF72 modulates STING-dependent inflammation by controlling its degradation (Figure 2H; McCauley et al., 2020). The accumulation of LAMP1 and the abnormal levels of cathepsin B in the macrophages and microglia of *C9orf72* KO mice confirm that the autophagy/lysosomal pathway is impaired in myeloid cells in the absence of C9ORF72 (Atanasio et al., 2016; Burberry et al., 2016; O’Rourke et al., 2016). Accordingly, C9ORF72 and SMCR8 double knockout mice present more severe immune defects than *C9orf72* KO only (Shao et al., 2020). When the autolysosomal pathway was studied specifically in myeloid cells from *C9orf72* KO, *Smcr8* KO, and double knockout mice, it was demonstrated that lysosomal activity was severely impaired due to the increased lysosomal exocytosis of mature lysosomal enzymes (Figure 2I; Zhang et al., 2018; Shao et al., 2020). Moreover, lysosomal dysfunction causes mechanistic target of rapamycin (mTOR) overexpression in those cells; interestingly, mTOR inhibition led to a rescue in macrophage dysfunction and peripheral immunity perturbation, including splenomegaly and lymphadenopathy. This recent study was the first to demonstrate the causal relationship between the role of C9ORF72 in the regulation of the autophagy/lysosomal pathway and the inflammatory effects resulting from C9ORF72 loss of function *in vivo* (Shao et al., 2020).

## C9Orf72 Haploinsufficiency in FTD/ALS

### The *C9ORF72* Mutation in FTD/ALS

#### A Non-Coding G_4_C_2_ Expansion in C9ORF72 Causes C9-FTD/ALS

When mutations in *C9ORF72* were discovered in familial cases of FTD and ALS in 2011, three independent teams provided evidence that the disease is caused by a (G_4_C_2_)*_n_* HRE G_4_C_2_ in the first intronic region of *C9ORF72* (Figure 2B; DeJesus-Hernandez et al., 2011; Renton et al., 2011; Gijselinck et al., 2016). This mutation with an autosomal-dominant transmission explains approximately 25% of familial FTD, 80% of cases with an FTD-ALS association, and 40% of familial ALS (van Blitterswijk et al., 2012; Majounie et al., 2012; Gijselinck et al., 2016). The size of the hexanucleotide expansion in healthy subjects ranges between 2 and 24, with most people harboring two to eight repeats. An expansion above 30 is considered pathological; a small proportion of patients present short expansions (30–100 units), while the vast majority have many hundreds or thousands of repeats (DeJesus-Hernandez et al., 2011; Gijselinck et al., 2012, 2016; van der Zee et al., 2013; Fournier et al., 2019). In addition to the heterogeneity of the repeat size between *C9ORF72* carriers, there is a mosaicism caused by the somatic instability of the repeat number in several tissues and among central nervous system regions (Van Mossevelde et al., 2017a). Thus, in the same patient, different expansion sizes can be measured in the CNS and in the blood (van Blitterswijk et al., 2013; Waite et al., 2014; Nordin et al., 2015). To establish correlations between repeat expansion size, age at onset, and disease severity, peripheral lymphocytes appear as an interesting non-invasive marker (Pamphlett et al., 2013). However, in addition to the mosaicism between tissues, the repeat number varies in blood with age at collection and over time in successive blood collections from *C9ORF72* mutation carriers (Suh et al., 2015; Fournier et al., 2019). This mosaicism and instability may be part of the mechanisms underlying the clinical heterogeneity observed in patients, but conclusive evidence is still lacking (Nordin et al., 2015; Van Mossevelde et al., 2017a). The fact that the expansion size increases with age in the blood could partly explain the conflicting results reported in the literature regarding age at onset and the size of the HRE (Beck et al., 2013; Dols-Icardo et al., 2014; Hübers et al., 2014; Waite et al., 2014; Suh et al., 2015; Chen et al., 2016; Gijselinck et al., 2016; Fournier et al., 2019; Jackson et al., 2020). Other usual features of pathological repeat expansions are also unclear in C9-FTD/ALS. One study reported decreased age at onset from one generation to the next, suggesting a mechanism of disease anticipation (Van Mossevelde et al., 2017b). Anticipation usually occurs when the size of the repeat increases in successive generations and is associated with increased disease severity. Overall, the HRE in *C9ORF72* appears to contract as much as it expands in blood during parent–child transmission (Gijselinck et al., 2016; Fournier et al., 2019; Jackson et al., 2020). Since familial and sporadic *C9ORF72* cases have nearly identical age at onset (Murphy et al., 2017)—but individual age at onset correlates with parental and mean family age at onset (Moore et al., 2020)—there are certainly a number of genetic and environmental modifiers interacting to determine the onset of symptoms.

#### Gain and Loss-of-Function Mechanisms

The non-coding HRE in *C9ORF72* has been shown to generatethree major pathogenic mechanisms: two related gain-of-functioneffects, concerning RNA and toxic protein aggregates, and one loss-of-function effect (Figure 1B). These mechanisms are intrinsically linked to transcription along the complex architecture of the *C9ORF72* locus. In variants 1 and 3, the HRE is located in the first intron as transcription includes exon 1a (Figures 2A,B). The repeated sequence of pure G and C forms highly stable DNA and RNA structures called G-quadruplex and R-loops (RNA–DNA hybrids; Figure 2B; Fratta et al., 2012; Reddy et al., 2013; Haeusler et al., 2014). In the HRE region, these structures cause the accumulation of aborted transcripts (Haeusler et al., 2014), and the structures formed by the expanded transcripts sequester the RNA-binding proteins involved in transcription and splicing. Consequently, these complexes called RNA foci affect posttranscriptional processing (Fratta et al., 2012; Lee et al., 2013). These RNA foci composed of both sense and antisense transcripts are a consistent feature in the brain tissue of *C9orf72* carriers (Lagier-Tourenne et al., 2013; Mizielinska et al., 2013). These foci can be found in the nuclei of the motor and frontal cortex neurons, hippocampus, cerebellum, and the spinal cord (Lagier-Tourenne et al., 2013; Mizielinska et al., 2013; Cooper-Knock et al., 2014). They are mainly observed in neurons, but can occasionally be detected in glial cells such as astrocytes, microglia, and oligodendrocytes (Lagier-Tourenne et al., 2013; Mizielinska et al., 2013). A second gain-of-function mechanism derives from the sense and antisense transcripts containing the HRE that induce ribosomes to translate them through a non-ATG (RAN) translation, resulting in the synthesis of dipeptide protein repeats (DPRs) that accumulate and form intracellular inclusions (Ash et al., 2013; Gendron et al., 2013; Mori et al., 2013; Zu et al., 2013). Inclusions of DPRs can be detected by immunohistochemistry, immunoblotting, or immunoassay. They are positive for p62 and negative for TDP-43 and may correspond to one or more dipeptides (Ash et al., 2013; Gendron et al., 2013; Mackenzie et al., 2013; Mori et al., 2013; Zu et al., 2013; Mackenzie et al., 2014). Inclusions of DPRs are mainly cytoplasmic, but can also be nuclear or found in dystrophic neurites or be present at the pre-inclusion stage disseminated in the cytoplasm (Mackenzie et al., 2015).

Finally, the presence of the repeat causes a downregulation in*C9ORF72* gene expression, leading to a heterozygous loss of function (LOF) (Figure 2B). A number of studies have explored why the mutation induces the haploinsufficiency of C9ORF72, focusing on epigenetic mechanisms. The repeat expansion in *C9ORF72* has been shown to bind to trimethylated histones in brain tissue from FTD/ALS patients (Belzil et al., 2013). Reduced messenger RNA (mRNA) levels of *C9ORF72* correlate with the trimethylation of these histone residues in patients’ frontal cortices and cerebella, while decreasing *C9ORF72* association with trimethylated histones restored *C9ORF72* mRNA expression in patients’ fibroblasts. Furthermore, the CpG island upstream of the pathogenic repeat and the repeat itself are hypermethylated (Belzil et al., 2014; Xi et al., 2014, 2015; Gijselinck et al., 2016; Jackson et al., 2020), and this hypermethylation correlates with repeat length and decreased promoter activity. While the methylation of the promoter and the expansion itself are now clearly associated with C9ORF72 haploinsufficiency, it can also result in a reduced accumulation of RNA foci and dipeptide repeat protein aggregates in human brains (Liu et al., 2014). Opposite effects on the disease onset, duration, and neurodegeneration have been reported, suggesting that epigenetic regulation of *C9ORF72* may be a potent pleiotropic disease modifier. Increasing methylation states have been associated with earlier onset ages (Gijselinck et al., 2016; Zhang et al., 2017), while *C9ORF72* promoter hypermethylation seems to slow disease progression and neurodegeneration (McMillan et al., 2015; Russ et al., 2015).

### C9ORF72 Expression in FTD/ALS Patients

Initial analyses of *C9ORF72* transcripts and protein levels have been relatively difficult and sometimes even misleading. The architecture of the gene and its transcripts have not been previously studied, making it difficult to get a real measure of the expression of the different variants, and commercially available antibodies for C9ORF72 have not been characterized (DeJesus-Hernandez et al., 2011; Renton et al., 2011; Gijselinck et al., 2012). Nevertheless, it is now firmly established that C9-FTD/ALS patients consistently present a decreased expression of *C9ORF72*.

#### Transcripts Expression

From the three variants resulting from *C9ORF72* transcription, V2 is by far the most abundant transcript in tissue from non-mutation carriers (van Blitterswijk et al., 2015; Tran et al., 2015; Rizzu et al., 2016). The HRE is located within the promoter region of this transcript, whereas it is in intron 1 of the other two transcripts, V1 and V3. Therefore, the epigenetic mechanisms discussed above, as well as the DNA G-quadruplex structures formed by the repeated GGGGCC, can variably interfere with the transcription of all three variants. While a global reduction of *C9ORF72* mRNAs, and more specifically of V2, was consistently observed since the discovery of the mutation (DeJesus-Hernandez et al., 2011; Gijselinck et al., 2012), the expressions of V1 and V3 in patients have been more debated. The total *C9ORF72* mRNA reduction was around 34% in lymphoblast cells from blood samples and was 38–50% in frontal cortex samples (DeJesus-Hernandez et al., 2011; Gijselinck et al., 2012). We observed a similar 50% decrease in frontal cortex and lymphoblasts from a group of patients in a French cohort (Ciura et al., 2013). A decrease in total *C9ORF72* mRNAs, and often also specifically of V2 and V1, was further reported in postmortem brain samples from C9-FTD/ALS patients, in particular in the frontal cortex and cerebellum (Belzil et al., 2013; Fratta et al., 2013; Waite et al., 2014; van Blitterswijk et al., 2015; Rizzu et al., 2016), as well as in the motor cortex and spinal cord (Donnelly et al., 2013). V3 appeared to be decreased (Fratta et al., 2013), unaffected (van Blitterswijk et al., 2015), or increased (Jackson et al., 2020). Sense and antisense transcripts containing the HRE were globally shown to be increased in the cerebellum of patients (Mori et al., 2013). Since V3 and V2 encode the same protein, and the part of V1 and V3 in the total fraction of *C9ORF72* mRNAs is minor, this will nevertheless not affect the general result that there is an overall decrease of ∼50% of *C9ORF72* transcripts and that C9ORF72 protein levels might be correspondingly reduced in C9-FTD/ALS patients. To date, no variation in the expression of total transcripts or variants 1, 2, and 3 was observed between FTD, ALS, and FTD-ALS patients (van Blitterswijk et al., 2015).

#### Protein Expression

Two C9ORF72 protein isoforms are known to exist in humans: a long isoform of 481 aa (C9-long of 54 kDa) encoded by V2 and V3 and a short isoform of 222 aa (C9-short of 24 kDa) encoded by V1. While C9-long appears well conserved in evolution, the short isoform is referenced only in humans and is not known in other non-human primates or other species (see Text Foot Note 1 for further details; Gene Tree for C9orf72 ENSG00000147894). Initially available commercial antibodies did not distinguish between the long and short isoforms; their specificity had not been strongly established and has been questioned since. A first study showed no differences in C9ORF72 protein expression in lymphoblast cells or brain lysates from FTD or ALS patients carrying the HRE compared to *C9ORF72* non-carrier individuals (DeJesus-Hernandez et al., 2011). Immunohistochemistry suggested a cytoplasmic staining in neurons with a punctate staining of gray matter, suggestive of synaptic localization. Another study found the protein to be predominantly localized within the nucleus and the C9ORF72 protein levels to be reduced in fibroblast cell lines derived from ALS patients relative to controls (Renton et al., 2011). In the following years, new antibodies have been developed that have revealed a clear decrease in C9ORF72 protein levels in the frontal cortex from FTD or ALS patients with *C9ORF72* repeat expansion compared to controls (Waite et al., 2014; Xiao et al., 2015; Saberi et al., 2018). This decrease ranges between 25 and 50% according to the studies and the employed antibodies. A similar or larger decrease was also reported in other parts of the cortex, in particular in the occipital, motor, and temporal cortices (Saberi et al., 2018; Xiao et al., 2015), while there was generally only a small (20%) (Frick et al., 2018) or no decrease in the cerebellum or spinal cord (Waite et al., 2014; Xiao et al., 2015; Saberi et al., 2018). In all cases but one, C9-short levels were too low to be detected, and therefore this reduction concerns only C9-long isoform. When C9-short could be detected, it appeared increased in the temporal and frontal cortices of C9-FTD/ALS patients (Xiao et al., 2015). This is in contradiction to the observed decrease in V1 transcript and bears no explanation so far, but it must be reminded that only three C9-ALS cases could be examined in that study and that the abundance of C9-short is generally admitted to be very low (Waite et al., 2014; Frick et al., 2018; Saberi et al., 2018). A sensitive and robust antibody-free mass spectrometry assay was recently used to measure the level of C9ORF72 isoforms, thus permitting to alleviate the antibody issue (Viodé et al., 2018). It was conducted on the frontal cortex of C9-FTD patients (with or without ALS) and demonstrated a decrease of 42% for total C9ORF72 and C9-long. C9-short-specific peptides were too low to be detected, confirming the scarcity of this isoform in human frontal cortex.

### Evidence of the C9ORF72 Haploinsufficiency Involvement in the Development of C9-FTD/ALS

Over the past decade, massive effort has been undertaken to decipher the mechanisms of C9-FTD/ALS pathogenesis. The three major hypotheses, toxicity of RNA foci or DPRs or C9ORF72 deleterious haploinsufficiency, have been investigated with some passion, and there is evidence for all three mechanisms. Many new experimental models have been constructed, and studies in animal models and human patient samples in particular, including iPSC-derived neurons, have provided unprecedented insights into the pathogenic mechanisms. The study of gain-of-function effects has benefited from the extensive knowledge already existing for other nucleotide repeat diseases (Braems et al., 2020). Because of the specific nature of the mutation and the complete absence of knowledge about the C9ORF72 protein, the loss-of-function effect was initially less explored. However, the tide has turned during the last 5 years, and not only C9ORF72 haploinsufficiency in C9-FTD/ALS patients is firmly established now, but its contribution to disease pathogenesis has been re-evaluated and has recently become unquestionable.

#### *In vivo* Loss-of-Function and Gain-of-Function Models

To better understand the consequences of C9ORF72 haploinsufficiency on FTD-ALS, numerous C9ORF72 loss-of-function *in vivo* models have been developed. In zebrafish and *Caenorhabditis elegans*, C9ORF72 loss of function resulted in locomotor phenotypes and motoneuron degeneration (Ciura et al., 2013; Therrien et al., 2013). Yet, the reduction of C9ORF72 in genetic mouse models or with antisense oligonucleotides did not mimic FTD-ALS (Balendra and Isaacs, 2018; Braems et al., 2020). Instead, reducing C9ORF72 to 40% with antisense oligonucleotide (ASO) resulted in an upregulation of the microglial activation genes in the spinal cord (Lagier-Tourenne et al., 2013), while *C9orf72* KO mice predominantly developed an inflammatory phenotype, sometimes associated with a shortened life span, but no neurodegeneration or motor neuron disease (Koppers et al., 2015; Atanasio et al., 2016; Burberry et al., 2016; Jiang et al., 2016; O’Rourke et al., 2016; Sudria-Lopez et al., 2016; Sullivan et al., 2016; Ugolino et al., 2016). Mild motor deficits were nevertheless noticed in two studies (Atanasio et al., 2016; Jiang et al., 2016), and although non-motor behavioral phenotypes were rarely assessed in *C9orf72* KO mice, early social recognition deficits were documented in one model (Jiang et al., 2016). These mammalian models made it clear that C9ORF72 deficiency is not the sole or more potent trigger of neurodegeneration in C9-FTD/ALS, but its exact contribution to the phenotype has not yet been fully understood. Pure gain-of-function mouse models, on the other hand, have also fallen short of recapitulating FTD/ALS. When overexpressing the HRE (Chew et al., 2015; Herranz-Martin et al., 2017) or specific DPRs (Zhang et al., 2016; Schludi et al., 2017; Choi et al., 2019; Hao et al., 2019; LaClair et al., 2020), both histopathology and some behavioral modifications related to FTD/ALS were observed. However, bacterial artificial chromosome (BAC) transgenic mice mainly reproduced RNA foci formation and DPR inclusions, whereas TDP-43 pathology, neurodegeneration, or FTD/ALS phenotypes were rare or inconstant (Peters et al., 2015; O’Rourke et al., 2016; Jiang et al., 2016; Liu Y. et al., 2016; Mordes et al., 2020; Nguyen et al., 2020). Spatial learning and anxiety abnormalities accompanied by loss of hippocampal neurons were observed by Jiang et al., but no motor deficits, neuromuscular unit alterations, or differences in social interaction, social recognition, or social communication (Jiang et al., 2016). Out of the four groups that generated C9ORF72 BAC mice, only one reported a progressive disease with a motor phenotype leading to hind limb paralysis associated with hippocampal and cortical neuron degeneration, though occurring only in a subset of mice (Liu Y. et al., 2016). Recently, the phenotype of these mice has been debated, as two different research groups did not succeed in reproducing these observations (Mordes et al., 2020) while two others did (Nguyen et al., 2020). An environmental factor was evoked since the experiments were carried out in different laboratories. Nonetheless, one of the major confounding effects could be the genetic background, as the used FVB/N background is known to present inherent seizure activity (Nguyen et al., 2020). Intriguingly, in the study of Mordes et al., even though DPR inclusions and C9ORF72 RNA foci were present at levels similar to those found in human C9-FTD/ALS brain, the mice did not show any phenotype. This supports the idea that the gain-of-function effect alone is not sufficient to induce neurodegeneration. Altogether, the gain-of-function models suggested that gain-of-function entities can cause neurodegeneration when sufficiently accumulated, but that additional insults are necessary when they are present at physiological levels.

#### Synergistic Models

In human iMNs derived from cells of C9-FTD/ALS patients, neurodegeneration was shown to depend on C9ORF72 haploinsufficiency that rendered iMNs hypersensitive to glutamate (Shi et al., 2018). Although little is known about the excitotoxicity mechanisms in FTD, in ALS, it is one of the major contributing mechanisms involved in the degeneration of motor neurons, and there is evidence that it is involved in C9-FTD/ALS (Starr and Sattler, 2018). An increase of glutamate receptors on the surface of iMNs was observed, leading to increased susceptibility to excitotoxicity. This neurodegenerative phenotype could be reproduced by deleting or decreasing C9ORF72 in control iMNs and could be fully rescued in C9-iMNs by re-expressing C9ORF72 (long or short). At the same time, the re-expression of C9ORF72 also reduced the accumulation of DPRs in C9-iMNs, which might have participated in rescuing the survival of C9ORF72 patient iMNs in response to glutamate treatment. Interestingly, a similar synergistic effect between C9ORF72 loss of function and excitotoxicity was very recently shown to cause ALS-like disease in rats (Dong et al., 2020a). KO rats did not spontaneously develop an ALS phenotype, but like the *C9orf72* KO mouse models, they developed splenomegaly and lymphadenopathy (O’Rourke et al., 2016; Dong et al., 2020a). When treated with a subclinical dose of kainic acid to promote glutamate excitotoxicity, motor deficits appeared in KO animals, associated with a loss of motor neurons (Dong et al., 2020a). Motor neurons also presented some abnormalities such as fragmented Golgi complex and abnormal vesicular trafficking. Moreover, Abo-Rady et al. showed a decreased axonal trafficking of lysosomes in iMNs derived from C9-FTD/ALS patient cells that was exacerbated by additionally knocking out *C9ORF72* in these cells (Abo-Rady et al., 2020). In this model, significant apoptosis of iMNs occurred only in cells presenting the combination of the HRE and the *C9ORF72* KO, while the knockout did not cause any effect by itself. These recent studies support the hypothesis that a total deletion of the C9ORF72 protein on its own is not sufficient to cause a motor phenotype, but it favors the appearance of the phenotype when combined with another defective or abnormal biological effect. In line with this, two other studies have now shown that the combination of gain-of-function mechanisms on top of the loss of function is efficiently leading to the development of an FTD/ALS-like disease in mice (Shao et al., 2019; Zhu et al., 2020). In both studies, reduction or loss of C9ORF72 provoked or exacerbated motor deficits in the background of C9-BAC mice. In the study by Zhu et al., they additionally documented that the same was true for cognitive deficits, neurodegeneration, glial activation, and accumulation of DPRs (Zhu et al., 2020). Finally, a knock-in (KI) model was made in rats demonstrating the need of combining loss- and gain-of-function effects to cause FTD/ALS (Dong et al., 2020b). Eighty G_4_C_2_ repeats with flanking fragments of human exons 1a and 1b were inserted in the rat *C9orf72* locus, resulting in a 40% decrease of C9ORF72 protein expression in the brain and spinal cord of KI rats. These rats developed a strong progressive motor deficit accompanied by a 47% loss of spinal motoneurons. Comparable phenotypes had never occurred in KO rats of the same genetic background or in BAC mice expressing larger HRE at a similar or a higher level. All these most recent models strongly argue in favor of a disease mechanism in C9-FTD/ALS resulting from reduced C9ORF72, synergizing with repeat-dependent gain of toxicity.

#### Patient-Based Evidence of the Contribution of C9ORF72 Haploinsufficiency to the Disease

Two strong arguments that are often invoked against a primary LOF mechanism in the disease concern the genetics of C9-FTD/ALS. Firstly, homozygosity does not strikingly increase the clinical severity of the disease, although only two cases were reported so far (Cooper-Knock et al., 2013; Fratta et al., 2013). Secondly, only the non-coding HRE and not simple LOF mutations in *C9ORF72* have been shown to cause C9-FTD/ALS (Harms et al., 2013). These are important arguments to consider, but they may not necessarily impede an essential role of the LOF in C9-FTD/ALS. The two homozygous cases are very different. The first one was a man with two large expansions inherited from his parents who had both developed early-onset dementia (Fratta et al., 2013). This patient presented a rapidly progressive severe dementia and died within 3 years after onset. The clinical and neuropathological characteristics of this homozygous patient are not beyond the range of the reported heterozygous cases, but nevertheless, they are at the most severe end of the spectrum. The second patient was a woman with one large expansion and a much shorter one of 50 ± 5 repeats (Cooper-Knock et al., 2013). The severity of her disease was unremarkable. Interestingly, the level of C9ORF72 transcripts was reduced by ± 75% in the frontal cortex of the first homozygous patient, while it was reduced to ± 50% of its normal level in the blood of the second patient, similarly to the average of heterozygous cases. It is worth noticing that, even in the case of two large expansions, there remains some C9ORF72 expression which is comparable to the lowest expression level that can sometimes be observed in heterozygous patients (Fratta et al., 2013; van Blitterswijk et al., 2015). Therefore, within these two cases, severity appears in agreement with the extent of C9ORF72 decrease, while remaining within the range of disease severity and partial loss of function that have been reported in cohorts of heterozygous patients. It is, however, not to be ignored that *C9ORF72* coding mutations are strikingly lacking in all C9-FTD/ALS cohorts that have been examined over the years. One splice mutation was reported in an ALS patient (Liu F. et al., 2016) from a Chinese cohort of 276 patients where mutations in 24 other ALS known causing genes had been excluded. This mutation resulted in a premature stop codon decreasing the level of the mutant messenger RNA, but the patient had no family history of the disease and autopsy could not be performed, which makes the interpretation of this case difficult. One might wonder if this mutation was not associated with a particular additional environmental trigger or to another variation in a potent modifier or causal gene that was not identified so far. Studies aiming to analyze whether the level of C9ORF72 associates with clinical characteristics in sufficiently large cohorts would greatly help in understanding the importance of haploinsufficiency in the disease. So far, one study conducted on 56 C9-FTD/ALS patients found a positive correlation between survival after onset and the level of V1 transcript in the frontal cortex and the cerebellum (van Blitterswijk et al., 2015). No further association with a clinical phenotype could be detected for the level of cerebellar C9ORF72 protein from 17 patients (Frick et al., 2018) or for the transcript levels in the blood of 75 C9ORF72 cases (Jackson et al., 2020). Interestingly, the C9ORF72 protein levels were consistently more reduced in the frontal cortex than in the cerebellum of patients (Waite et al., 2014; Xiao et al., 2015; Saberi et al., 2018), highlighting the possibility that it will be important to quantify C9ORF72 transcripts and protein specifically from the affected brain regions to see whether it correlates with some clinical characteristics. The case reported by McGoldrick et al. was of particular interest in this regard (McGoldrick et al., 2018). Siblings severely affected by ALS and carrying a long expansion in *C9ORF72* had inherited it from their mosaic father (unaffected at age 90) who carried a 70-repeat allele in blood but larger expansions in the CNS. Remarkably, the father never developed the disease or TDP-43 pathology, but he had similar RNA foci and DPR burdens compared to his affected daughter, while increased expression and protein level of C9ORF72 could be detected in his blood and CNS tissues. Considering all the evidence now gained from the various *in vitro* and *in vivo* models, it seems more and more established that C9-FTD/ALS pathogenesis needs the combination of C9ORF72 haploinsufficiency and the accumulation of toxic DPRs or expanded RNA. Maybe the question should now be considered differently: could *C9ORF72* loss-of-function mutations be pathogenic by themselves or are they more likely to be a risk factor? Whether one or the other, in which type of conditions are they likely to play a role? Considering the role of C9ORF72 in autophagy and immune regulation, as well as its function to regulate the inflammatory reaction to gut microbiota, maybe coding LOF mutations are more likely to be found in patients suffering from the dysregulation of these functions? For example, LOF mutations could be explored in systemic lupus erythematosus and rheumatoid arthritis, where it was recently shown that there is an increased proportion of *C9ORF72* intermediate expansions (Fredi et al., 2019). Alternatively, as the pro-inflammatory effects of *C9ORF72* LOF lead to increased antitumor activity in mice (McCauley et al., 2020), it would be interesting to investigate whether *C9ORF72* germinal or somatic variants can be associated with differences in survival or disease evolution in patients suffering from glioma or other types of cancers. Interestingly, there is evidence of decreased incidence of some cancers (Fang et al., 2013; Gibson et al., 2016) and overrepresentation of autoimmune disorders in patients with ALS (Miller et al., 2013, 2016; Turner et al., 2013). In particular, patients with C9-FTD/ALS present an abnormally high prevalence of inflammatory arthritides, cutaneous conditions, and gastrointestinal disorders (Miller et al., 2016). Finally, further evidence that C9-FTD/ALS patients bear the mark of C9ORF72 haploinsufficiency and that its pathological consequences are part of the disease comes from neuropathological examination and RNA sequencing studies. *C9orf72* KO mice, *C9ORF72* patients iPSC-derived, and *C9ORF72*^±^ iMNs share similar gene expression changes to the postmortem tissue and blood from C9-FTD/ALS patients, in particular regarding the immune and endolysosomal pathways (O’Rourke et al., 2016; Shi et al., 2018; McCauley et al., 2020). Reactive microglia are frequent in the spinal cords of ALS cases, but microglia with large accumulations of lysosomes were only observed in the C9-ALS cases (O’Rourke et al., 2016), strengthening the causal link between immune dysregulation and endolysosomal impairment in the pathogenesis. Moreover, ubiquitin- and p62-positive inclusion bodies are characteristic of C9-FTD/ALS cases (Al-Sarraj et al., 2011; Mackenzie et al., 2014), supporting the strong involvement of the autophagy pathway in the disease. Recently, machine learning applied to RNA sequencing from the frontal cortex of 34 *C9ORF72* expansion carriers identified the vesicular transport module as a potent modifier of survival after onset (Dickson et al., 2019), while neuronal transcriptome of the mid-frontal neocortex from seven postmortem *C9ORF72* expansion carriers showed that global C9-FTD/ALS-associated transcriptome changes appear driven by the loss of function of the C9ORF72 protein (Liu et al., 2020).

## Conclusion

Increasing knowledge about the functions of C9ORF72 has revealed that this protein is part of a GTPase-interacting complex which is a potent regulator at the crossroad of autophagy and inflammation. Interestingly, C9ORF72 also emerges as a new protein linking neurodegeneration, inflammation, and the regulation of our immune interaction with the environment. Remarkably, there is considerable genetic evidence linking the autophagy/lysosomal pathway to FTD/ALS (Deng et al., 2017; Stamatakou et al., 2020), as proteins encoded by *SQSTM1*, *OPTN*, *TBK1*, *VCP*, *UBQLN2*, and *CHMP2B*, all genes involved in ALS and FTD, also function in this pathway. This and the generation of new complex models, as well as deeper analysis of patients’ data, have contributed to re-evaluating the role of C9ORF72 haploinsufficiency in the disease. It must now be considered when developing therapies that C9-FTD/ALS results from the detrimental combination of impaired cellular homeostasis caused by C9ORF72 partial loss of function and the accumulation of toxic gain-of-function entities (expanded RNAs, DPRs, or both). While several other strategies targeting the various mechanisms of the disease are currently under study (Balendra and Isaacs, 2018; Malik and Wiedau, 2020; Ghasemi et al., 2021), ASO therapies are clearly the most advanced, with an ongoing phase I trial of BIIB078 lead by Biogen (clinicaltrials.gov Identifier: NCT03626012). BIIB078 selectively targets *C9ORF72* transcript variants 1 and 3 that carry the expansion. At first, while the contribution of the loss of function to the disease seemed uncertain, ASOs depleting all *C9ORF72* transcripts or selectively expansion-containing transcripts were developed in parallel (Donnelly et al., 2013; Sareen et al., 2013; Lagier-Tourenne et al., 2013; Jiang et al., 2016; Gendron et al., 2017). Now, the importance of preserving C9ORF72 protein expression has been emphasized in a new ASO development (Liu et al., 2021), but strategies aiming to restore control levels of the C9ORF72 protein are still missing.

## Author Contributions

JS and ML wrote the manuscript. E-GB designed the figures and edited the manuscript. All authors contributed to the article and approved the submitted version.

## Conflict of Interest

The authors declare that the research was conducted in the absence of any commercial or financial relationships that could be construed as a potential conflict of interest.

## Footnotes

1 www.ensembl.org
